# Virologic Nonsuppression Among Patients With HIV Newly Diagnosed With Cancer at Uganda Cancer Institute: A Cross-Sectional Study

**DOI:** 10.1200/GO.22.00262

**Published:** 2023-04-12

**Authors:** Geraldine Kauma, Henry Ddungu, Isaac Ssewanyana, Sharon Nyesiga, Naghib Bogere, Teddy Namulema-Diiro, Pauline Byakika-Kibwika, Elizabeth Namukwaya, Harriet Mayanja Kizza

**Affiliations:** ^1^ Uganda Cancer Institute, Kampala, Uganda; ^2^ Uganda Central Public Health Laboratory, Kampala, Uganda; ^3^ Uganda Heart Institute, Kampala, Uganda; ^4^ Victorville Clinic Gayaza, Wakiso, Uganda; ^5^ Department of Medicine, School of Medicine, College of Health Sciences, Makerere University, Kampala, Uganda; ^6^ Infectious Diseases Institute, College of Health Sciences, Makerere University, Kampala, Uganda

## Abstract

AIDS-related mortality declined markedly since the introduction of antiretroviral therapy (ART); however, cancer mortality in Africa was higher than its incidence in 2020. People living with HIV (PLWHIV) are at an increased risk of malignancy and death from malignancy compared with the general population. In Uganda, AIDS-defining malignancies (ADMs), including cervical cancer, Kaposi sarcoma, and non-Hodgkin lymphoma, are among the commonest malignancies. Virologic nonsuppression has been identified as an important predictor of mortality among PLWHIV diagnosed with cancer. This study aimed to determine the prevalence and to identify factors associated with virologic nonsuppression among PLWHIV newly diagnosed with cancer.

**METHODS:**

This was a cross-sectional study that was carried out between December 2018 and April 2019 at the Uganda Cancer Institute. PLWHIV who had been on ART for at least 6 months and were newly diagnosed with cancer were enrolled.

**RESULTS:**

A total of 167 participants were enrolled. Cervical cancer was the commonest ADM (n = 45; 50.6%) of all ADMs, while esophageal and breast cancers were the commonest non-ADMs, accounting for 17.5% (n = 14) each of all non-ADMs. The prevalence of virologic nonsuppression was 15%. Having Kaposi sarcoma (odds ratio [OR], 8.15; *P* = .003), being poorly adherent to ART (OR, 4.1; *P* = .045), and being on second-line ART (OR, 5.68; *P* = .011) were associated with virologic nonsuppression.

**CONCLUSION:**

The prevalence of virologic nonsuppression is high among patients with HIV newly diagnosed with cancer. These findings emphasize the need for strengthening of adherence strategies, optimizing ART regimens, and prioritization of viral load testing among PLWHIV with newly diagnosed malignancy.

## INTRODUCTION

According to the Joint United Nations Programme on HIV/AIDS (UNAIDS), 38.4 million people had HIV globally in 2021, with an incidence of 1.5 million people and mortality of 650,000 people; the prevalence of HIV in Eastern and Southern Africa was 20.6 million, with an incidence of 670,000 and 280,000 deaths reported. The AIDS-related mortality on the basis of the same report is said to have reduced by 68% in 2004 and by 52% since 2010 mainly because of usage of antiretroviral therapy (ART).^1^ In 2020-2021, the prevalence of HIV among adults in Uganda was 5.8%, with an annual incidence of 0.29%.^2^

**CONTEXT**

**Key Objective**
What is the prevalence and factors associated with virologic nonsuppression among patients with HIV newly diagnosed with cancer?
**Knowledge Generated**
Virologic nonsuppression has been assessed previously among patients with HIV without specific consideration for patients with HIV coinfected with cancer. The prevalence of virologic nonsuppression among patients with HIV newly diagnosed with cancer was found to be 15%, associated with having Kaposi sarcoma, being poorly adherent on ART, and being on second-line ART.
**Relevance**
Initiation and optimization of ART through viral load monitoring among people living with HIV diagnosed with cancer alongside cancer therapy is paramount for better survival among this special group of patients.

In 2020, the incidence of cancer was 19.3 million, with 10 million deaths worldwide. In Africa, the incidence of cancer was found to be 5.7%, a value lower than recorded cancer deaths, which was found to be 7.2%.^3^ The incidence of cancer in Uganda was 34,008 and cancer deaths were 22,992, with the commonest cancers being cervical cancer, Kaposi sarcoma, breast cancer, prostate cancer, and non-Hodgkin lymphoma.^4^

The reduction in the above-mentioned HIV-related deaths described is not reflected among patients with HIV who develop cancer. In fact, evidence shows that cancer is one of the commonest causes of morbidity and mortality among patients with HIV, contributing almost a third of the deaths.^5^

People living with HIV (PLWHIV) have an elevated risk for many cancers, partly due to HIV-related immunosuppression and other cancer risk factors, for example, smoking and alcohol use.^6^ AIDS-defining malignancies, which include Kaposi sarcoma, non-Hodgkin lymphoma, and cervical cancer, accounted for most cancer-related morbidity and mortality among patients with HIV in the pre-highly active antiretroviral therapy era. Although current evidence suggests a significant decline in the rates of AIDS-defining malignancies (ADMs), the absolute numbers are still high.^7^ HIV-infected individuals are still at a higher risk of developing non-ADMs (NADMs) in comparison with the general population.^8,9^ Further still, the risk of death in the year after a cancer diagnosis is more than twofold among PLWHIV compared with their HIV-negative controls.^10^

While managing PLWHIV coinfected with cancer, monitoring their response to ART is necessary. Even so, CD4 T-cell count is not an appropriate measure as it is affected by either the malignancy or cancer treatment.^11^ Virologic suppression is recognized as an important predictor of survival among patients with HIV and cancer.^12,13^ Evidence from a study done by Gopal et al^14^ among PLWHIV diagnosed with lymphoma showed that patients with higher HIV viral loads for 6 months after lymphoma diagnosis had worse survival with a 35% increase in mortality.

Initiating ART among ART-naive patients and optimizing the ART regimens for patients already on therapy is recognized as an important strategy associated with improved survival among patients with HIV and cancer.^15,16^ It is important that we understand the factors that are associated with virologic nonsuppression to identify those PLWHIV newly diagnosed with cancer at risk of poor survival. These patients can be prioritized for closer monitoring to improve treatment outcomes.

## METHODS

### Study Design and Setting

This was a cross-sectional study that was carried out between December 2018 and April 2019 at the Uganda Cancer Institute (UCI), the national cancer referral center, located in Kampala, Uganda. We enrolled patients who were newly diagnosed with cancer and were HIV-positive on ART for at least 6 months.

### Ethical Considerations

The School of Medicine Research and Ethics Committee (REC REF 2019-165) approved the study. Informed consent was sought and obtained from all participants. Administrative clearance was sought from the Uganda Cancer Institute Research Ethics Committee. All participants were assigned study identification numbers without the use of names.

### Study Population and Patient Recruitment

We screened all patients with HIV who had been on ART for at least 6 months, attending the UCI with a documented cancer diagnosis confirmed on histology or cytopathology, age 18 years and older, and ability to provide written informed consent to participate in the study. We excluded patients who were unable to ascertain information pertaining to their HIV disease status or ART. Information on the patient demographics and comorbidities were obtained from patients using a short-structured questionnaire. Clinical information on HIV disease WHO stage, ART regimen and duration on treatment, and most recent CD4 for the patient was obtained through medical records from their primary HIV care center.

Cancer-related information, including histology diagnosis, staging, and treatment, were obtained from patient files at UCI.

### Blood Collection and Viral Load Testing

Under aseptic technique, 4 mL of blood was drawn from patients and placed into EDTA Vacutainer tubes. The samples were taken to the UCI laboratory within 30 minutes from the time they had been drawn off, centrifuged at 3,000 revolutions per minute to separate the plasma and blood cells, and stored at 4°C before transport to Uganda Central Public Health Laboratory, the national public health reference laboratory, for viral load testing. HIV viral load was estimated using Abbott m2000rt Real-Time HIV assay, and test accuracy has been reported at 95% at the 1,000 copies/mL cutoff with a sensitivity and specificity of 96.6% (95% CI, 91.8 to 98.7) and 90.4% (95% CI, 78.2 to 96.4), respectively, in several clinical settings.^17^ To detect HIV-1 RNA ≥400 copies/mL, the test sensitivity, specificity, positive predictive value, and negative predictive value were 91.8%, 100%, 100%, and 92.2%, respectively.^18^

### Study Variables

*Dependent variable*: viral load of > 1,000 copies on plasma as measured within at least 6 months of starting ART and upon enrollment into the study. This was based on current recommendation in Uganda.^4^

**FIG A1:**
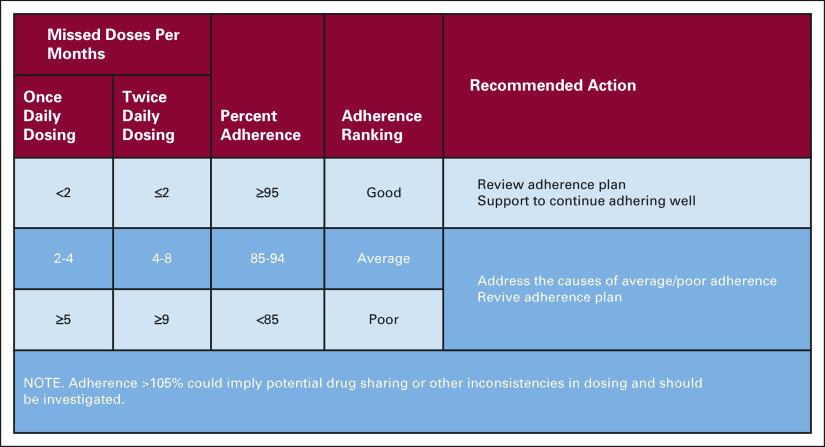
Table for determining ART adherence level (extracted from consolidated HIV guidelines 2020). ART, antiretroviral therapy.

*The independent variables* included patient factors (age, sex, marital status, education level, smoking, and alcohol use), HIV disease factors (duration with diagnosis, stage of disease, CD4 count, and HIV viral load), and HIV treatment factors (ART regimen; whether first, second, or third line on the basis of local treatment guidelines, duration of treatment, and adherence to treatment); this was based on self-report by patients, and responses to questions including (number of drugs taken daily, how many doses were missed in past month and reasons for missing doses) were entered into the tool extracted from the Uganda National Guidelines for prevention and treatment of HIV (Appendix Fig A1).^4^ Other independent variables included comorbidities (hypertension, diabetes mellitus, viral hepatitis and tuberculosis) and cancer factors (type of cancer and stage of cancer; which is staged as I through IV for most cancers, while Kaposi sarcoma is staged as low risk or high risk).

### Data Management and Analysis

Data were entered in Epi-Data Version 3.2 and then exported to Stata software V14.0 (Stata Corp, College Station, TX) for analysis. It was checked for errors, cleaned, and coded before analyzing. Continuous variables were analyzed using means, medians, and standard deviations, whereas frequencies, proportions, and percentages were used for categorical variables. The outcome variable was viral load nonsuppression measured within at least 6 months of enrollment into study. Participants were categorized as either suppressed or nonsuppressed (defined as a viral load of at least 1,000 copies of viral RNA/mL on plasma). Chi-square test or Fisher's exact tests were used to make comparisons across groups of categorical variables as appropriate while comparing prevalence of special groups. Logistic regression was used to determine the association between the independent variables and viral load nonsuppression. Bivariate analysis was performed to determine how each of independent variable was independently associated with the outcome. Only variables with *P* values < .2 as well as some factors that have previously been found to be associated with virologic nonsuppression but were not significant in our bivariate analysis were included in the multivariate model. In the multivariate model, *P* values < .05 were considered statistically significant in explaining the associations between the independent variables and viral load nonsuppression.

## RESULTS

**FIG A2:**
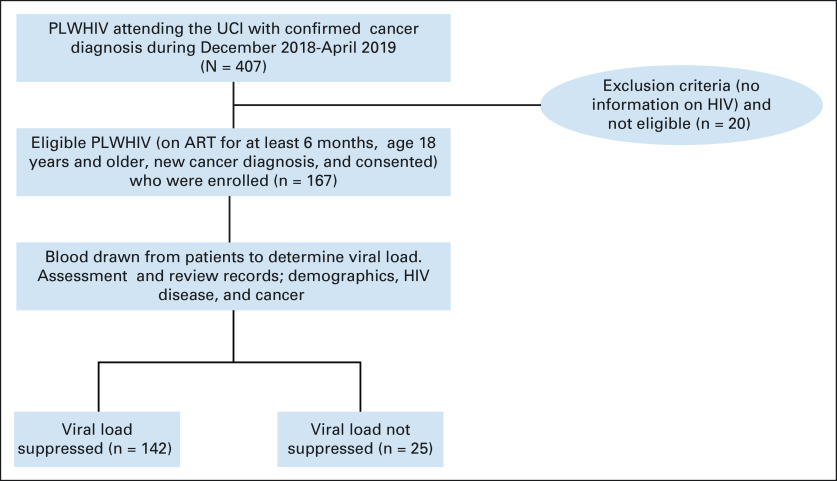
Patient flow diagram. ART, antiretroviral therapy; PLWHIV, people living with HIV; UCI, Uganda Cancer Institute.

We enrolled 167 patients (Appendix Fig A2), and their baseline characteristics are summarized Table 1 below.

**TABLE 1:**
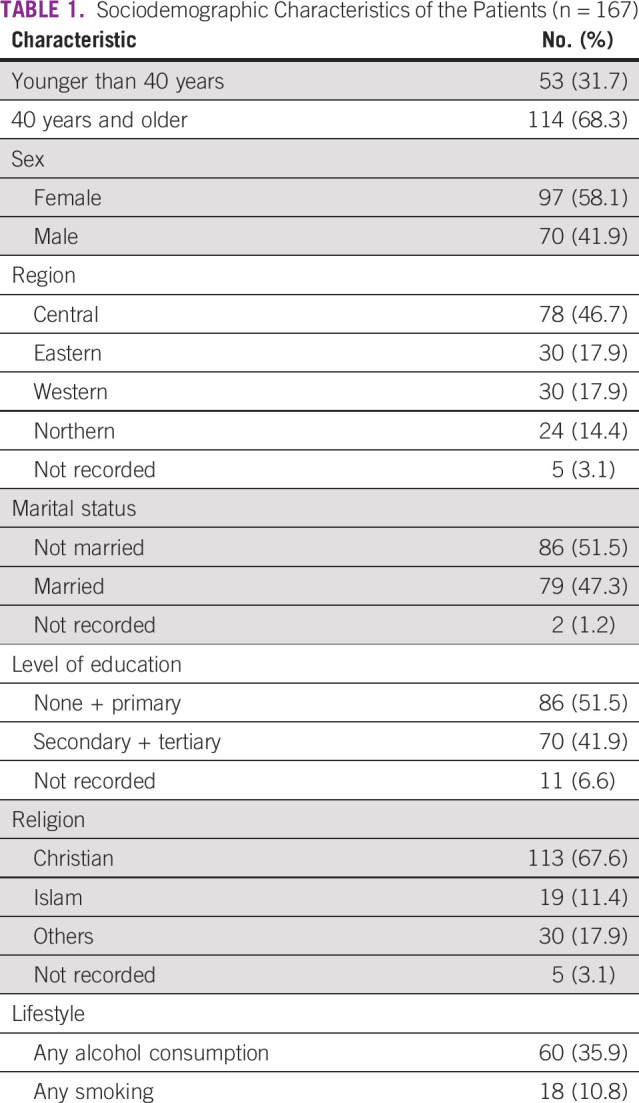
Sociodemographic Characteristics of the Patients (n = 167)

### Sociodemographic and Clinical Characteristics

Among the 167 patients recruited in our study, 97 (58.1%) were female and 114 (68.3%) were older than 40 years. Majority had good adherence to ART, 113 (74.8%). Of the 129 (77.2%) patients who had their cancer stage determined, 62% had advanced cancer (stage 3 or 4) and poor risk stage for Kaposi sarcoma (Table 2).

**TABLE 2:**
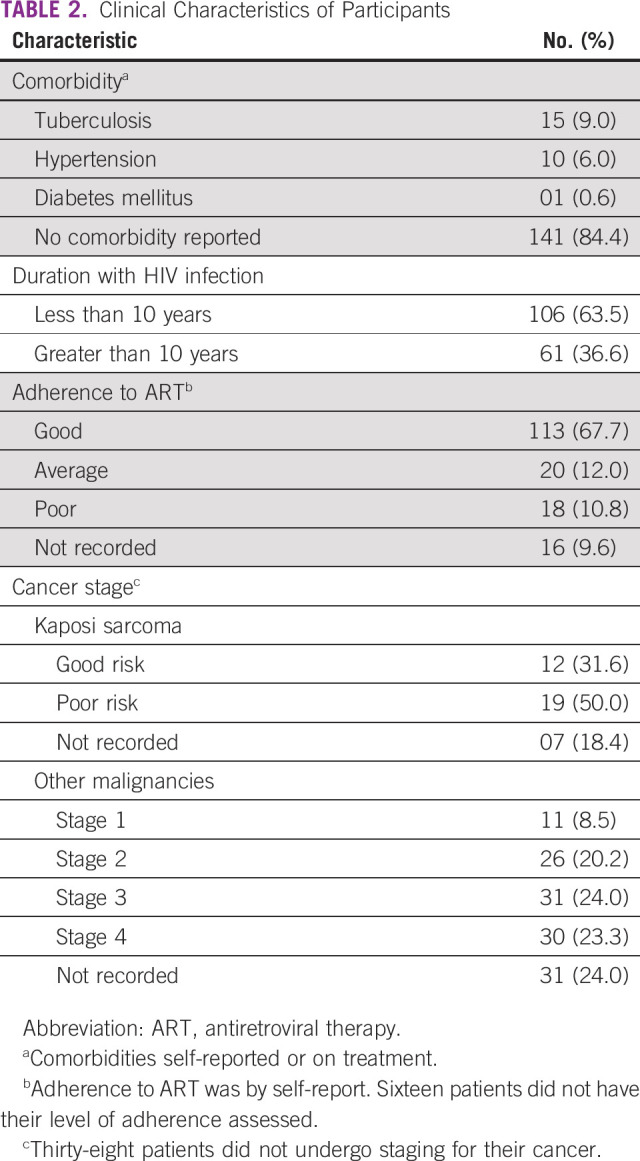
Clinical Characteristics of Participants

### Cancer Diagnosis Among the Patients

**FIG 1:**
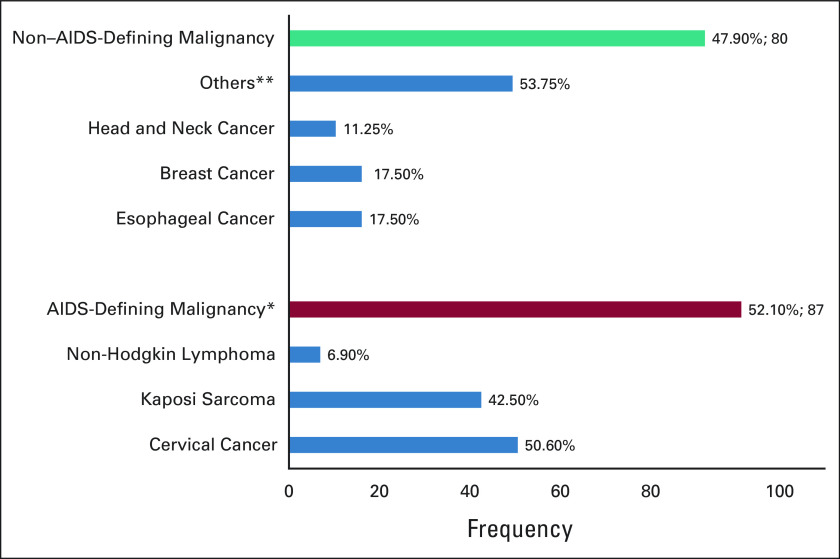
Cancer type distribution among the patients. ^*^AIDS-defining malignancy: cancer in which patients with HIV are at higher risk of acquiring compared with uninfected people of the same age, and include Kaposi sarcoma, cervical cancer, and lymphoma. ^**^Others: Hodgkin lymphoma, sarcoma, anaplastic carcinoma, cancer of conjunctiva, cancer of epiglottis, cancer of larynx, prostate cancer, cancer of vulva, gastrointestinal stromal tumor, cancer of hypopharynx, oral pharyngeal cancer, postcricoid cancer, squamous cell carcinoma of eye, cancer of palate, spindle cell intra-abdominal neoplasm, multicentric Castleman's disease, and penile cancer.

Eighty-seven (52.1%) of the patients had ADMs, while 80 (47.9%) had NADMs. Cervical cancer was the commonest ADM, accounting for 45 (50.6%) patients with ADMs, while Kaposi sarcoma accounted for 38 (42.5%) patients with ADMs. Esophageal cancer and breast cancer were the commonest NADMs, each accounting for 14 (17.5%) patients with NADMs (Fig 1).

### Prevalence of Viral Load Nonsuppression Among Patients With HIV Newly Diagnosed With Cancer

The prevalence of viral load nonsuppression among patients with HIV newly diagnosed with cancer was 15% (95% CI, 10.3 to 21.3), as shown in Table 3.

**TABLE 3:**
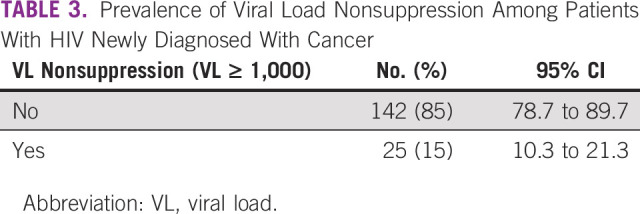
Prevalence of Viral Load Nonsuppression Among Patients With HIV Newly Diagnosed With Cancer

### Factors Associated With Virologic Nonsuppression Among the Patients

At bivariate analysis, the factors that were significantly associated with viral nonsuppression included being diagnosed with Kaposi sarcoma, poor adherence to ART, and being on second-line ART. Being married was associated with less odds of being nonsuppressed (Table 4).

**TABLE 4:**
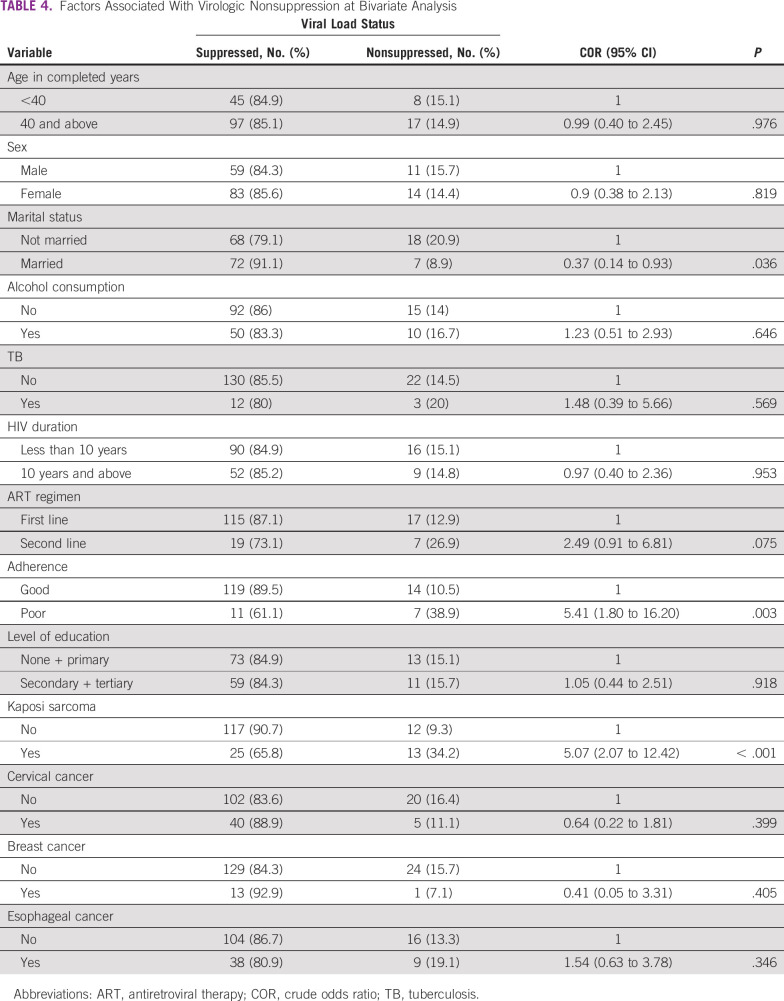
Factors Associated With Virologic Nonsuppression at Bivariate Analysis

At multivariate analysis, patients with Kaposi sarcoma (odds ratio [OR], 8.15; 95% CI, 2.00 to 33.22; *P* = .003), those with poor adherence to ART (OR, 4.1; 95% CI, 1.03 to 16.28; *P* = .045), and those on second-line ART (OR, 5.68; 95% CI, 1.48 to 21.84; *P* = .011) were more likely to be nonsuppressed (Table 5).

**TABLE 5:**
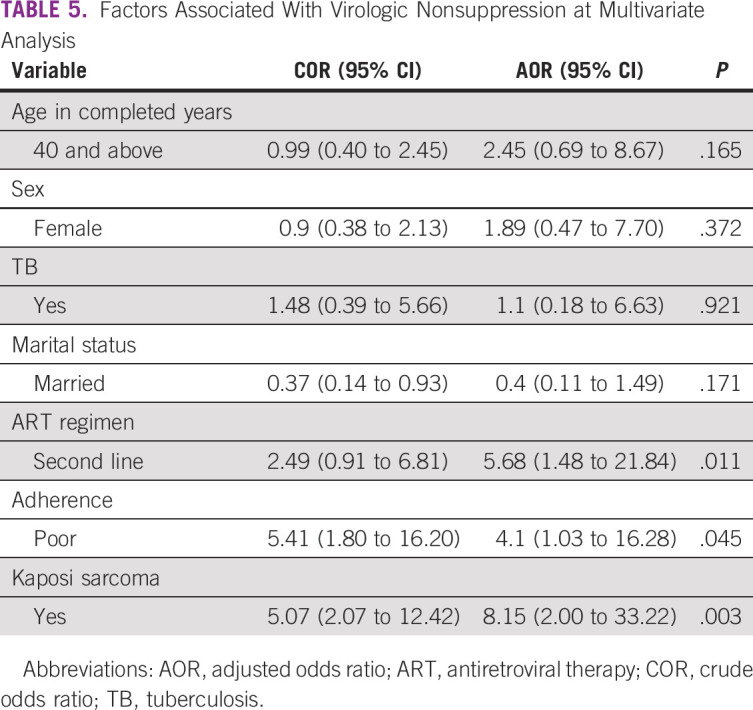
Factors Associated With Virologic Nonsuppression at Multivariate Analysis

## DISCUSSION

In this cross-sectional study, the prevalence of virologic nonsuppression among patients with HIV who were ART-experienced at cancer diagnosis was 15%. The factors that associated with virologic nonsuppression among these patients included having a diagnosis of Kaposi sarcoma, poor adherence to ART, and being on second-line ART.

The current ART coverage in Uganda according to UNAIDS 2021 report is 82%. ART regimens comprise two nucleoside reverse transcriptase inhibitors (NRTIs), the backbones, and a third drug or anchor from another class that can be either integrase strand transfer inhibitors, Protease Inhibitors (PI), or non-NRTIs. The preferred first line ART regimen is a combination of Tenofovir (TDF) + Lamuvidine (3TC) + Dolutegravir (DTG), altenative regimens include; TDF + 3TC + Efavirenz (EFV); Abacavir (ABC) + 3TC + DTV or EFV; TDF + 3TC + Atazanavir/ritonavir (ATV/r); ABC + 3TC + ATV/r, HIV care in Uganda is funded through Ugandan Ministry of Health, multilateral donations including the President's Emergency Plan for AIDS in Africa, and Global Fund and other international funders, and includes free at point of delivery or out-of-pocket financing and health insurance provided at private for-profit and not-for-profit facilities.^19^

The prevalence of virologic nonsuppression in our study is slightly higher than the 11% found in a study done by Bulage et al,^20^ who used routinely collected data of all PLWHIV from all health facilities in Uganda. This highlights that the prevalence of virologic nonsuppression is higher in PLWHIV newly diagnosed with cancer than the average of all patients with HIV. A study done in Vietnam among patients with HIV attending four public clinics found a prevalence of viral load suppression of 93%, which could imply 7% of the study population were virologically nonsuppressed. This is much lower than our finding of 15%.^21^ Cancer development and progression are associated with chronic inflammation and immunosuppression, both seen in HIV infection.^6^ Majority of our patients also had advanced stage at diagnosis, usually associated with more frail symptoms such as vomiting and dysphagia, which can hinder adherence and cause virologic nonsuppression.

Our study found HIV virologic nonsuppression to be associated with having Kaposi sarcoma (OR, 10.89; 95% CI, 2.17 to 54.71; *P* = .004). Uncontrolled HIV viral replication is associated with an increased risk of developing AIDS-defining malignancies.^22-24^ The Kaposi sarcoma human herpesvirus 8 and HIV interact in various ways; the HIV Tat-protein causes growth of Kaposi sarcoma (KS) infected cells by stimulating the proliferation of spindle cells. It also activates cytokine genes such as interleukin-6 and inhibits apoptosis by interferon-gamma. The same Tat protein is also directly angiogenic, increasing infectivity of the human herpesvirus 8. This is all compounded by the HIV disease itself, which is inflammatory and associated with increased inflammatory cytokines (interleukin-1 and interleukin-6, among others) that also promote angiogenesis. The immune suppression caused favors replication of human herpesvirus 8, a known infectious cause of the KS.^25^ In fact, effective ART, which causes suppression of HIV by reducing viral replication, is helpful in controlling KS.^26^

Our study also found an association between adherence to ART and virologic nonsuppression (OR, 4.1; 95% CI, 1.03 to 16.28; *P* = .045). Adherence to ART is known to result in virologic nonsuppression, and this is not unique to noncancer patients. Emphasis on optimizing ART adherence to reduce HIV viral loads and the risk of acquiring cancer has previously been demonstrated.^27,28^

Many cancers are associated with nonspecific gastrointestinal symptoms, including anorexia, nausea, and vomiting.^29^ These symptoms could result in reduced drug intake resulting in previously suppressed patients becoming nonsuppressed. Other cancers such as esophageal cancer, intestinal Kaposi sarcoma, and stomach cancer directly cause difficult or painful swallowing. These symptoms can affect patients' ability to swallow ART drugs and thus adherence. Patients enrolled in our study gave some similar reasons for missing drugs, specifically including the inability to swallow the drugs because of cancer symptoms such as difficulty in swallowing, being too ill and weak to swallow medication, awaiting referral to a cancer treatment center, and not getting ART drug refills.

Another possibility is some of these patients were nonsuppressed even before development of cancer and at the time of diagnosis and measurement of viral loads. A possible cause of virologic nonsuppression could be viral blips, which are transient elevations in viral load that do not require ART switch. Blips can occur because of low viral replication that eventually can result in virologic failure.^30^

Our study also found virologic nonsuppression to be associated with being on second-line antiretroviral treatment (OR, 9.86; 95% CI, 1.68 to 57.77; *P* = .011). A plausible explanation for the above association could be because the patients had accumulated resistant mutations hence failure to achieve suppression subsequent lines of treatment. This was elaborated by Pujades-Rodríguez et al^31^ who showed that poorly adherent noncancer patients were three times more likely to fail second-line therapy. Depression is common in both cancer and HIV.^29,32^ Having both diagnoses increases the patient likelihood of suffering symptoms of depression. This may affect a patient likelihood of taking medication even after a switch to second-line ART. The current guidelines recommend two NRTIs in combination with ritonavir-boosted PI as second-line therapy.^4^ In the case of lopinavir being used, the patient faces the burden of taking drugs twice a day, which has intolerable gastrointestinal effects such as diarrhea, nausea, and vomiting. In addition to the cancer-related symptoms, these side effects are likely to affect adherence to ART. Nonetheless, during data analysis for factors associated with virologic nonsuppression, our study did not take into account the duration patients had been on second-line therapy.

Several authors have described younger age to be associated with virologic nonsuppression. This group of patients faces barriers to counseling since it is indirect through the caregiver. There are also challenges associated with peer stigma, sexual and reproductive health, and disclosure, and all these contribute to inadequate adherence.^33,34^ In our study, patients enrolled were mainly adults, with the majority being older than 40 years. This could have been a possible reason why age was not found to be associated with virologic nonsuppression. However, a higher percentage of patients older than 40 years were nonsuppressed but with no significant association on logistic regression.

In our study, we did not assess the relationship between CD4 count and viral load nonsuppression because most of the patients in our study did not have CD4 counts done. This can be explained by the fact that current national ART guidelines do not recommend routine CD4 count measurements for patients who are stable on ART,^4^ although several studies have found low CD4 cell counts to be associated with virologic nonsuppression.^34-36^ An exception to this occurs in elite controllers with high T-cell activation resulting in CD4 cell loss with low viremia.

The relationship between cancer stage and viral load nonsuppression was not assessed in this study. This was because a high proportion of patients were not staged; some died before staging, and others were lost to follow-up. However, previous studies that included patients with HIV and non–HIV-infected patients showed no relationship between cancer stage and HIV diagnosis.^37^ Achenbach et al^12^ acknowledged the association between increased risk for mortality among patients with HIV with nonsuppressed viral loads, noting the role of HIV replication in tumor pathogenesis and growth. This could possibly imply advanced stage is associated with higher viral loads, thus an area worth further research.

Some limitations of our study included the study being conducted at the UCI, a specialized referral center for patients with cancer, which has no HIV clinic. Follow-up of patients' HIV treatment and level of adherence was difficult. Written consultation notes were sent for patients with nonsuppressed viral loads to notify primary ART care providers about the results to carry out the appropriate intervention. Adherence was assessed on the basis of patient self-report, which was found to be comparable with pill counts by Haberer et al^38^; despite this, self-report is subject to bias since patients may not recall, which is another limitation. A more recent study showed a less positive correlation with hair drug concentration when compared with pill counts and pharmacy refills.^39^ Several studies have reported no gold standard for measuring ART adherence, with most having advantages and disadvantages.^39-41^ Finally, the study had a component of review of records, which was affected by missing information because of inadequate documentation. The study sample size attained was small over a limited period of time, which can affect external validity of the results, but still giving some vital data in this special group of patients.

In conclusion, in this study, we found a high prevalence of virologic nonsuppression among patients with HIV with cancer and specifically associated with having a diagnosis of Kaposi sarcoma, having poor adherence to ART, and being on second-line treatment. Therefore, this specific group of patients, among others, needs routine viral load tests at diagnosis and intense follow-up to make necessary ART drug adjustments and possibly enhance adherence to ART treatment. From the study findings, we recommend routine HIV viral load tests for PLWHIV newly diagnosed with cancer at the UCI. This would ensure necessary interventions such as ART drug adjustments or adherence counseling be done even before starting chemotherapy.
